# Impact of 25 Years of Mobile Health Tools for Pain Management in Patients With Chronic Musculoskeletal Pain: Systematic Review

**DOI:** 10.2196/59358

**Published:** 2024-08-16

**Authors:** Jenny Lin-Hong Shi, Regina Wing-Shan Sit

**Affiliations:** 1 Department of Medicine Jockey Club School of Public Health and Primary Care Prince of Wales Hospital, The Chinese University of Hong Kong Hong Kong China

**Keywords:** mHealth, mobile health, mobile app, chronic musculoskeletal pain, pain management, patient compliance, adherence, usability, feasibility, acceptability, PRISMA

## Abstract

**Background:**

Mobile technologies are increasingly being used in health care and public health practice for patient communication, monitoring, and education. Mobile health (mHealth) tools have also been used to facilitate adherence to chronic musculoskeletal pain (CMP) management, which is critical to achieving improved pain outcomes, quality of life, and cost-effective health care.

**Objective:**

The aim of this systematic review was to evaluate the 25-year trend of the literature on the adherence, usability, feasibility, and acceptability of mHealth interventions in CMP management among patients and health care providers.

**Methods:**

We searched the PubMed, Cochrane CENTRAL, MEDLINE, EMBASE, and Web of Science databases for studies assessing the role of mHealth in CMP management from January 1999 to December 2023. Outcomes of interest included the effect of mHealth interventions on patient adherence; pain-specific clinical outcomes after the intervention; and the usability, feasibility, and acceptability of mHealth tools and platforms in chronic pain management among target end users.

**Results:**

A total of 89 articles (26,429 participants) were included in the systematic review. Mobile apps were the most commonly used mHealth tools (78/89, 88%) among the included studies, followed by mobile app plus monitor (5/89, 6%), mobile app plus wearable sensor (4/89, 4%), and web-based mobile app plus monitor (1/89, 1%). Usability, feasibility, and acceptability or patient preferences for mHealth interventions were assessed in 26% (23/89) of the studies and observed to be generally high. Overall, 30% (27/89) of the studies used a randomized controlled trial (RCT), cohort, or pilot design to assess the impact of the mHealth intervention on patients’ adherence, with significant improvements (all *P*<.05) observed in 93% (25/27) of these studies. Significant (judged at *P*<.05) between-group differences were reported in 27 of the 29 (93%) RCTs that measured the effect of mHealth on CMP-specific clinical outcomes.

**Conclusions:**

There is great potential for mHealth tools to better facilitate adherence to CMP management, and the current evidence supporting their effectiveness is generally high. Further research should focus on the cost-effectiveness of mHealth interventions for better incorporating these tools into health care practices.

**Trial Registration:**

International Prospective Register of Systematic Reviews (PROSPERO) CRD42024524634; https://www.crd.york.ac.uk/prospero/display_record.php?RecordID=524634

## Introduction

Chronic musculoskeletal pain (CMP) is defined as musculoskeletal pain that persists or recurs for longer than 3 months [1]. Interventions to help manage CMP are usually based on multimodal and biopsychosocial models [2-4]. CMP is a global burden, affecting approximately 1 in 5 adults [5]. One study indicated that over 70% of people 65 years and older have experienced an episode of joint pain [6]. Given that the percentage of the population 65 years and older is expected to increase from 15% to 24% by 2060, chronic musculoskeletal conditions will definitely become an increasing burden for the health care system [7].

The long-term nature and frequent need for continuous monitoring in CMP management gave rise to early developments in telemonitoring and telehealth. These innovations designed to improve CMP management and prevent disability and death have been improved by ongoing technological advancements. One such advancement is mobile device–based health care, or mobile health (mHealth). In 2022, the number of mobile users worldwide stood at 7.26 billion, which is projected to reach 7.49 billion by 2025 [8]. Mobile technologies such as smartphones and wireless monitoring devices are increasingly finding innovative applications and have emerged as potential alternatives to support the self-management of patients with CMP [9]. These applications include communication, data collection, patient monitoring, and education, as well as to facilitate adherence to CMP management [9].

The available evidence has pointed to the promising effects of mHealth interventions on CMP. A recent review [10] evaluated the effectiveness of app-based interventions on several CMP conditions (including general chronic pain, osteoarthritis [OA] pain, chronic neck pain [CNP], chronic low back pain [CLBP], rheumatoid arthritis, menstrual pain, migraine-related pain, and frozen shoulder pain), stating that mobile apps are significantly more effective in reducing pain compared with control conditions. Du et al [11] analyzed the use of web-based and mHealth interventions in patients with CLBP, showing that mHealth tools had a better effect on both pain and functional outcomes. In a similar vein, Thurnheer et al [12] analyzed the efficacy of mobile app usage in the management of patients with cancer and noncancer pain (eg, acute pain, general chronic pain, CNP, CLBP, and menstrual pain), reporting beneficial effects on pain relief, particularly in the out-clinic setting.

The evidence of the use of mHealth systems is still emerging, mainly focusing on its effect on clinical outcomes. However, current CMP management often requires a long-term care plan. Adherence to CMP management is critical to achieving improved outcomes, quality of life (QoL), and cost-effective health care [13]. A review of adherence behaviors from the World Health Organization noted that “increasing adherence may have greater effects on health than improvements with specific medical therapy” [14]. The true impact of these mHealth tools on adherence to treatment regimens may be overlooked, as mHealth promoters are more eager to show their effects on clinical outcomes (eg, morbidity, mortality, and biometric markers of clinical disease). Adherence to CMP treatment is a critical link that would connect the promise of mHealth to the ultimate goal of improving clinical outcomes.

To the best of our knowledge, no review published to date has examined the effects of using mHealth tools on the adherence to management and clinical outcomes of patients with CMP. Therefore, the aim of this systematic review was to provide an overview of the evidence with respect to a broad range of outcomes, including feasibility, usability, acceptability, and adherence, of mHealth tools to impact CMP-specific clinical outcomes. This approach enabled considering mHealth tools at all stages of development and to gauge the effectiveness of these tools across a range of technologies and CMP subtypes, many of which have overlapping treatment regimens and require similar adherence behaviors.

## Methods

### Overview

The protocol of this systematic review was registered on the International Prospective Register of Systematic Reviews (PROSPERO) database (CRD42024524634). The review was performed following the 2020 PRISMA (Preferred Reporting Items for Systematic Reviews and Meta-Analyses) guidelines [15].

We undertook a 25-year systematic review of mHealth interventions used to facilitate the self-management of CMP and adherence to treatment and management regimens. For this review, CMP included all types of chronic primary and secondary musculoskeletal pain based on the complete list of CMP conditions in the *International Classification of Diseases, 11th edition* (ICD-11) foundation layer supplement [16]. Our definition of mHealth was adopted from the Global Observatory for eHealth definition: “medical and public health practice supported by mobile devices” [17]. Given the comprehensive nature of CMP, this review goes beyond defining adherence as compliance with a treatment regimen by including a wide range of interventions such as medication reminders, symptom monitoring, educational tools, and facilitated patient-provider communication [18].

### Search Strategy

The search strategy was based on CMP diseases according to the ICD-11 [19]. The search was conducted from January 1999 through December 2023. Using Boolean phrases, we searched the PubMed, MEDLINE, Cochrane (CENTRAL), EMBASE, and Web of Science databases for studies that assessed the role of mHealth interventions in CMP management. The search strategy was first developed for the PubMed database using Medical Subject Headings (MeSH) and was adapted for other databases. The search was filtered either by English language or by date of publication or publication type as an article. The detailed search strategy for each database is provided in Multimedia Appendix 1.

### Inclusion Criteria

We included original research published in peer-reviewed journals that evaluated the effects of mHealth tools on CMP-related clinical outcomes; adherence to management; and usability, feasibility, and acceptability features. All available study designs (randomized controlled trials [RCTs], cohort studies, cross-sectional studies, case series, case reports, questionnaires, mixed methods studies, and qualitative studies) were eligible for inclusion. Allowing for flexibility in the outcomes measured and in study design was necessary for an inclusive view of mHealth interventions at all stages of design, development, and evaluation. The detailed inclusion criteria for study selection and mHealth tools are provided in Multimedia Appendix 2.

### Exclusion Criteria

Only articles reporting mHealth interventions designed for CMP were included. We excluded articles regarding interventions that were not tested in a sample population with clearly described methods and results. In addition, review articles, editorials, commentaries, dissertations, poster presentations, abstracts, proposals for future studies, study protocols, and descriptive articles describing new tools but not testing them in a sample population were excluded. The publication language was restricted to English.

### Data Extraction and Analysis

Publications were initially screened for potential inclusion based on a simultaneous review of titles and abstracts by 2 independent reviewers. Any discrepancies were resolved by consensus among the researchers. Information, including publication year, location, study sample characteristics, types of mobile technology used, intervention and control details (if available for the given study design), outcomes measured, and summary results reported, was extracted and compiled using a Microsoft Excel spreadsheet.

We performed descriptive analyses of the data and summarized the findings from the included studies with an emphasis on statistical results reported in RCTs and cohort studies. We highlighted differences between groups when these results were available. Studies were organized for analysis based on the primary objective of the study and the key outcomes measured. Outcomes were organized into the following categories: (1) usability, feasibility, and acceptability of the mHealth tool; (2) effect of the mHealth intervention on adherence to CMP management; and (3) effect of the mHealth intervention on pain outcomes.

## Results

### Summary

In all, 866 articles were retrieved in full text and assessed for eligibility, among which 383 articles were excluded due to duplication. Based on the search criteria, 337 articles were excluded due to not meeting the study design criteria or not aligning with the definition of mHealth used in our study. A total of 142 articles were included in the full-text retrieval process, of which 53 were excluded due to an inappropriate study design, intervention, or population or no access to the full text. Finally, a total of 89 articles were eligible and met all inclusion criteria; the details of these included studies are provided in Multimedia Appendix 3 [20-107]. Multimedia Appendix 4 illustrates the PRISMA flowchart for the study selection process and Multimedia Appendix 5 provides a list of excluded studies with reasons for exclusion.

### Study Characteristics

**Figure 1:**
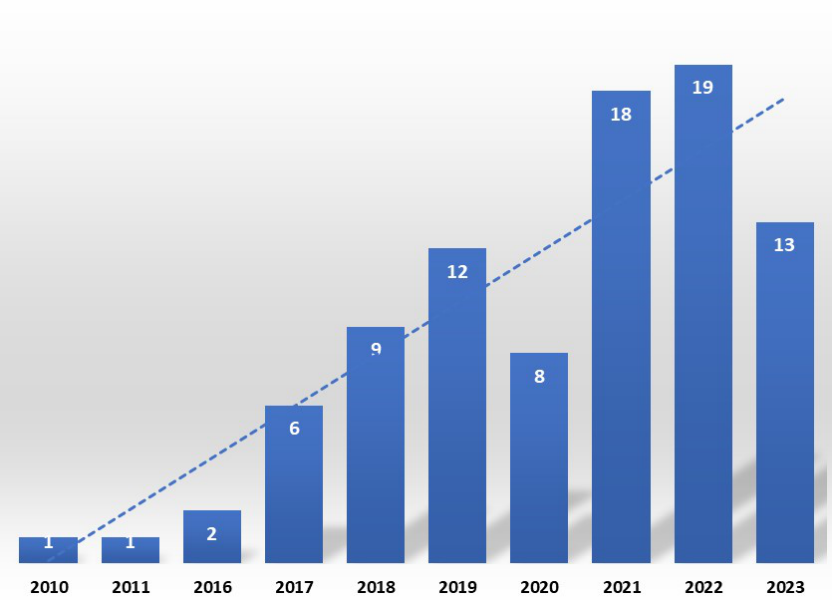
Trend in publication number from 1999 to 2024.

**Figure 2:**
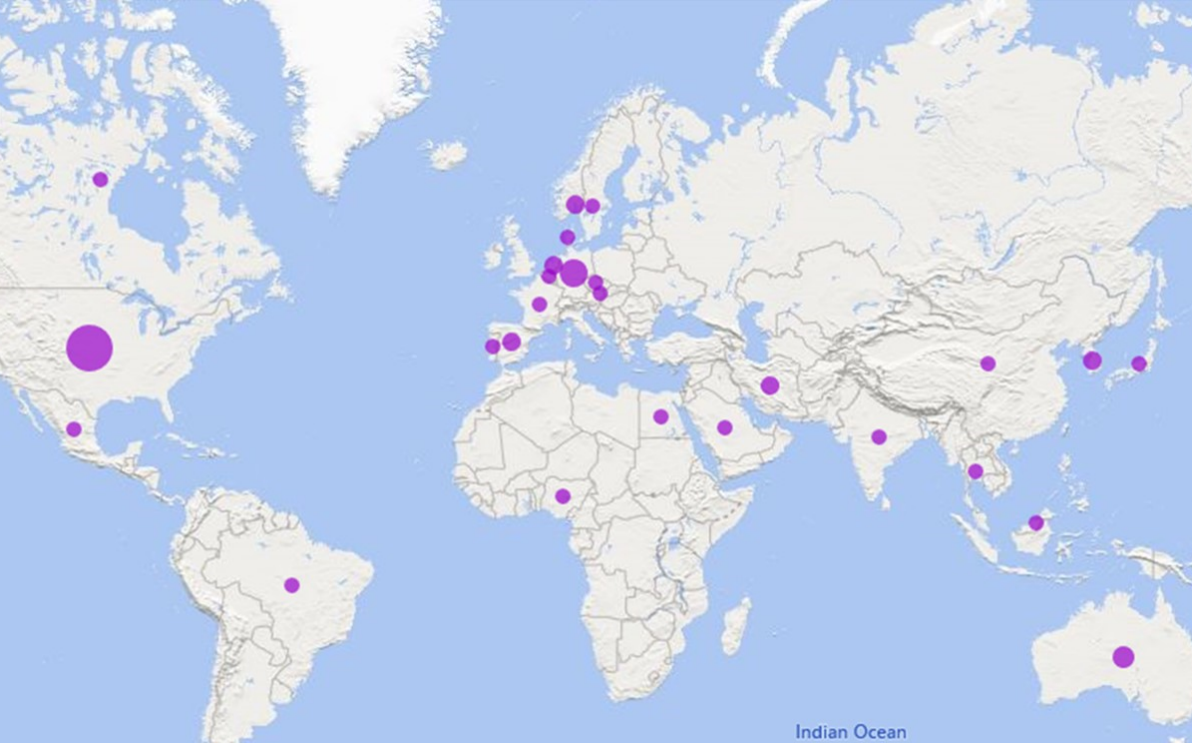
Eligible studies published by country. The size of the purple circles indicates the number of publications, with larger circles reflecting a higher number.

**Figure 3:**
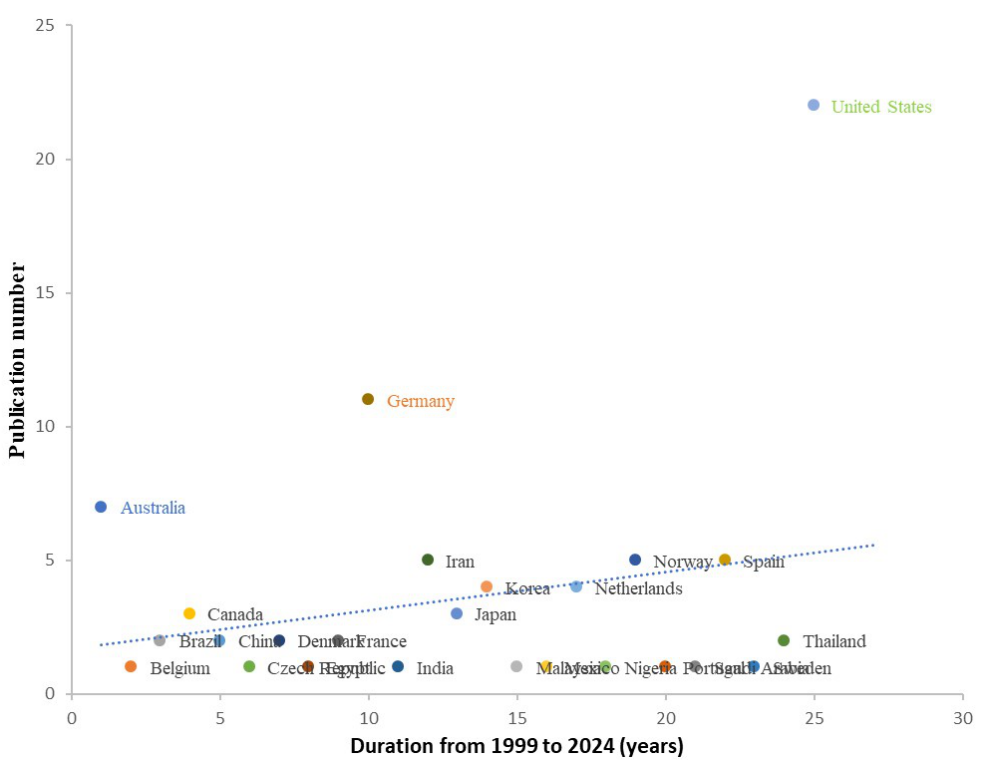
Publications from different countries from 1999 to 2023.

The publication time period for this 25-year systematic review spanned from 1999 to 2023, with an overall increase in articles published after 2017 and a significant increase during the COVID-19 pandemic from 2019 to 2022 (Figure 1). A total of 26 countries published eligible studies across 5 continents (Asia, Europe, Africa, America, and Oceania) from 1999 to 2023. Most of the eligible studies were published in Europe (Figure 2). The top-3 ranked countries publishing in this field were the United States (22/89, 25%), Germany (11/89, 12%), and Australia (7/89, 8%) (Figure 3). The vast majority of technology development for mHealth has been based in the United States, especially in the earlier days of smartphone introduction.

RCTs that assessed the differences between different mHealth tools or between an mHealth tool and standard or control care were the most common study designs, accounting for 35% (31/89) of the included studies, followed by cohort (23/89, 26%), pilot (14/89, 16%), cross-sectional (8/89, 8%), qualitative (7/89, 8%), and questionnaire (4/89, 4%) study designs; there was only 1 (1%) case series, case report, and cost-effectiveness study each. Study durations ranged from only a few hours to 12 months depending on the study design.

### mHealth Users

The CMP populations considered in this review included adult and young patients with nonspecific CMP, specific OA without or with surgery, CLBP, and neck pain. Patients with OA and CLBP were the most commonly investigated groups among the included studies. The characteristics of the target user group were often the key impetus for the development of the mHealth tool. For example, some researchers noted that a certain percentage of patients report suboptimal satisfaction for various reasons after total knee arthroplasty, such as continued pain or stiffness [108-111]. For patients with knee pain 24 months post triple arthrodesis following a crush injury, a specific mobile camera could immediately provide corrective techniques when applying in-clinic equipment to the patient’s shoe [102]. We identified 10 studies addressing the perceptions or experiences about mHealth tools in adult CMP populations with and without surgery [26,28,30,55,62,85,87,95,112] and their practitioners [30,35,87], as well as in young people [90]; the authors of these studies agreed that mHealth interventions were acceptable, useful, and feasible. However, there is still a gap between patients and physicians in understanding and communication about the treatment and management of knee OA [30]. It is expected that multiple stages of user experience testing could be a template for future mHealth tools aimed at chronic disease management [113]. However, only 1 eligible article [87] mentioned an mHealth app prototype that was codeveloped with patients and health care providers (HCPs) in 2021. Ultimately, it appears that diverse individuals can use mHealth tools as long as the tools are tailored to the needs of the population and sufficient training and support are provided.

### Types of Tools Used in mHealth for CMP

In most of the reported mHealth interventions, mobile phones or other devices were either provided to users or considered a required intervention for study participation. We classified the studied mHealth tools into 4 main categories: mobile app, mobile app plus monitor, mobile app plus wearable sensor, and web-based mobile app plus monitor. The details of each type of mHealth tool are provided in Multimedia Appendix 6. Mobile app (78/89, 88%) was the most commonly used tool and the primary platform for patients with CMP. For example, mHealth apps could be installed on the patient’s mobile phone at any time to help remember to check pain symptoms, maintain physical therapy, connect to coaches, or communicate virtually with HCPs in real time. The next most common mHealth tool reported was a mobile app plus monitor, which was used in 6% (5/89) of the included studies, followed by a mobile app plus wearable sensor (4/89, 4%) and web-based mobile app plus monitor (1/89, 1%). One study reported multiple digital technologies. These mHealth programs focused mainly on a combination of mobile and internal or external monitors or an external sensor, thus facilitating the transfer of data automatically without requiring the patient to manually submit the data and providing faster delivery of self-management therapy.

### Study Outcomes

#### Overview

Multiple outcome measures were used to evaluate mHealth tools depending on diverse study objectives. For the purposes of this analysis, the outcomes were organized into 3 categories: (1) usability, feasibility, and acceptability; (2) effect of the mHealth intervention on adherence to CMP management protocols; and (3) effect of the mHealth intervention on clinical outcomes.

#### Impact on Adherence

A total of 30 of the 89 studies (34%) evaluated or reported the effect of an mHealth intervention on adherence to CMP management, including medication adherence, engagement in healthy intervention therapy, and frequency of symptom monitoring. These studies include 2 questionnaire studies, 1 case series, 10 cohorts, 9 pilot studies, and 8 RCTs with or without a control group. Among these 30 studies, 1 (3%) observed mixed results, 25 (83%) showed a significant difference (judged at *P*<.05), and 1 (3%) found no difference. Multimedia Appendix 7 provides an overview of these studies [29,35,38,41,42,49,52-58,61,65,68,71,72,75, 80,81,84,91,92,98-101,104,106].

Mobile apps with a monitor or wearable sensor were mainly investigated in patients with OA, which were used as daily reminders for self-management. Studies in patients with OA [80] and those undergoing surgery showed significant improvements of the apps with respect to patient adherence rates [38,49,65,100]. Some studies observed that a mobile app combined with a motion sensor or monitor could support early rehabilitation with good compliance [65], suggesting that surgeons can consider these two tools as appropriate alternatives to traditional physical therapy programs after joint surgery [49,100]. For younger patients with CMP, leveraging extant digital tools with appropriate user-informed adaptations can also help to build capacity tailored to support young people’s self-management of musculoskeletal pain [90]. For other CMP groups, such as patients with CLBP and CNP, use of a mobile app alone was the main intervention. Although recognizing the inadequacy of traditional neck pain treatments compared with treating CLBP, a mobile app implemented with a self-classification algorithm was found to be particularly effective in increasing adherence to an exercise program among older and younger office workers with neck pain [58]. However, another study indicated that the clinical importance of added adherence with use of a mobile app is unclear in a specific population with upper- or lower-limb musculoskeletal conditions [56]. Often, using an mHealth system as an interface between the patient and the provider was perceived as less burdensome and associated with less judgment compared to face-to-face contact, particularly in situations in which the patients were not fully adherent to the recommended treatment [87,112]. mHealth tools facilitated better management and improved patient confidence to monitor CMP, making the patients feel in control and strengthening their coping mechanisms.

#### Impact on Clinical Outcomes

A total of 55 of the 89 included studies (62%) assessed or reported the effect of mHealth on disease-specific clinical outcomes, including pain intensity, pain-related function, pain-related disability, pain-related cognition, health-related QoL, and medication use. These studies include 1 case series, 13 cohort studies, 4 cross-sectional studies, 7 pilot studies, 1 questionnaire study, and 29 RCTs. Of the 29 RCTs that measured the effect of mHealth on CMP-specific clinical outcomes, 28 (93%) reported significant differences (judged at *P*<.05) between groups; no significant differences were found in 1 (3%) study and mixed results were observed in 1 (3%) study. In addition, a significant effect of mHealth tools was observed in 13 cohort studies and 7 pilot studies. Multimedia Appendix 8 provides an overview of these studies [20-23,25,27,29,34,37,39-42,44-48,50,51,53,54,56,58,59, 61,63,64,67-69,72-74,76,78,79,81-83,86,88, 89,94,96-101,103-107].

A total of 49 interventions (including RCTs, cohort studies, and pilot studies) were related to improving pain intensity outcomes. Among these 49 studies, 46 (94%) reported significant improvements in pain intensity. Both younger and older patients receiving app messages with tailored instructions on pain management experienced statistically significant improvements in their pain intensity levels compared to those of patients receiving usual care or an intervention without an mHealth tool. However, mobile app–based relaxation exercises did not effectively reduce CNP [68], highlighting the importance of future mHealth tools to include an individualized and tailored program. Another trial did not show a significant improvement in pain perception at 6 months, although the mHealth tool tested in this trial was determined to be feasible and associated with a satisfactory user experience [53]. Among the outcomes of the mHealth tools evaluated, 26 studies focused on pain-related functional performance, 15 studies focused on pain-related disability, 15 studies focused on pain-related cognitive performance, 14 studies focused on pain-related QoL, and 4 studies focused on pain-related medication use. Generally, mHealth tools were associated with a significant improvement in functional and cognitive performance and QoL, along with a significant decrease in the disability burden and medication use. However, in patients with CLBP, the improvement in pain-related disability was small and of uncertain clinical significance after using a self-management app for 9 months [82].

### Usability, Feasibility, and Acceptability

Among the 89 included studies, 23 (26%) assessed or reported usability, feasibility, and acceptability using qualitative methods and compiled usage data, including 1 case series, 9 cohort studies, 4 cross-sectional studies, 6 pilot studies, 1 qualitative study, 1 questionnaire study, and 1 RCT. These data ranged from patient satisfaction to cost-effectiveness estimations as well as the timing and frequency of engagement with mobile apps and platforms. Multimedia Appendix 9 provides an overview of these studies [22,24,29,33,38,43,45,49,52,57, 61,66,71,72,74,77,80,92,93,95,101,107].

In general, these studies found mHealth tools and platforms to be usable, feasible, acceptable, and appreciated among users compared with traditional measures. For example, both older and younger patients with CMP and those who underwent joint surgery perceived that using an mHealth tool increased their independence and confidence in pain management [22,38,80]. One study reported that the prescription of therapeutic exercises via a smartphone app is feasible and well-accepted among patients of all ages [112]. Seven studies showed the long-term (>3-month follow-up) feasibility and acceptability of mHealth tools [24,33,38,49,52,72,101]. Specifically, with long-term follow-up, patients with knee or hip OA seemed to have preferences for goals related to physical activity and nutrition rather than for goals related to vitality and education [71]. Patients with OA undergoing primary hip or knee arthroplasty particularly appreciated the mHealth tools empowering patients, facilitating transitions from specialized hospital care to primary care, reducing unplanned contacts with the health system, and reducing overall health costs, proving to be cost-effective [38]. Another study performed in a real-world setting with a large (N=10,264) and diverse population experiencing CMP found that mHealth was accepted and considered especially useful for pain reduction [62]. The majority of studies included in this review focused on the patient as the end user of mHealth tools, although some also evaluated acceptability and perceptions from the perspective of HCPs. Features of mHealth tools such as automated reminders, messages with educational and motivational content, healthy living challenges, and wireless transmission of data contributed to increased self-care awareness and knowledge about CMP.

## Discussion

### Main Findings

This review showed that interventions based on mHealth systems have beneficial effects on adherence and clinical outcomes for individuals with CMP. Thus, this scientific evidence suggests that these mHealth systems could be promising alternatives for CMP self-management through multimodal approaches. The evidence presented here indicates that while the potential of mHealth tools is high, their results during implementation and execution are nevertheless mixed.

Mobile apps are the most widely reported mHealth tool for interventions, which have been successfully used to facilitate adherence to CMP management and improve clinical outcomes [114,115]. The freedom and portability of mobile devices combined with the advanced capacity to facilitate 2-way communication and collect and analyze data for a real-time response offer enormous potential to both patients and providers [116,117]. Owing to their abilities for automation, personalization, and easy integration into existing health systems, mobile apps are less operator-dependent and are less reliant on processes to facilitate the active and time-consuming exchange of information compared to traditional tools. However, apps may have a limitation in terms of difficulty of use for the older population with minimal technology experience or familiarity, and there is clearly room for improvement. Moreover, there is a lack of scientific and health professional support in many available mHealth systems, highlighting the need for developing appropriate apps based on the well-recognized guidelines in the management of CMP [113].

There is a growing recognition of the need for digital technologies to improve access to age-appropriate resources and personalized support for cocare [118-120]. Unlike previous reviews in this field that only included RCTs or cohort studies, this systematic review also included 13 qualitative studies (including questionnaire, interview, and discussion studies). These informative studies used qualitative methods that yielded rich data that can be used to better understand how and why mHealth tools impact adherence behaviors and clinical outcomes. Qualitative data can also enable patient-physician discussions regarding modifiable self-management options based on the perspectives and needs of patients, HCPs, or both groups. Moreover, user feedback can inform hypotheses that can then be tested. Research that seeks to understand how and why mHealth works will deliver on the broader promise of mHealth. Future mHealth tools will be able to draw on the knowledge generated when discrete hypotheses around the relative importance of, for example, patient-provider communication, optimal user interfaces, or targeted motivational messages are tested. These informative studies could lead to better mHealth tools that deliver improved health outcomes.

### Implications and Future Directions

This review found that the usability, feasibility, and acceptability of mHealth tools for CMP management and adherence to different programs were generally high among both patients and providers. mHealth offers a way to address different barriers to care and to reduce health disparities from both patient and HCP perspectives. However, only one article published in this field over the last 25 years mentioned that the mHealth app prototype was codeveloped by patients and HCPs [87]. There will be more opportunities to codesign mHealth tools in the future. Undoubtedly, innovative mHealth tools could unintentionally increase health disparities due to unequal access to technology. There is also recognition that unequal access to, use of, and knowledge of information can influence the uptake and use of mHealth tools. These inequalities and the needs from target user groups should be taken into consideration early in the design and development of future mHealth tools. However, none of the studies included in this review addressed systematic differences in usability between diverse patient groups and geographical areas. Future research can be designed to better understand these differences and to encourage the development of mHealth tools that address the needs of diverse patient groups and populations living in regions with different levels of economic development.

The high prevalence of CMP globally coupled with the advantages of providing help through apps offers opportunities to help countless people who may be looking to the potential of mHealth to lessen the burden of their pain. One key aspect of this potential involves an increase in cost-effectiveness and expanded outreach of pain management. Of note, only one study included in the review specifically focused on the issue of cost-effectiveness [60]. Furthermore, only one study presented the postmarketing observational data after a follow-up duration of 9 months [96]. Rigorous cost-effectiveness analyses will be necessary to demonstrate not only the health impact but also the value of investing in these innovations. Future studies evaluating the cost-effectiveness of mHealth tools are indeed needed, since we can see that pockets of mHealth innovations are expanding around the globe (Figure 3). Besides cost, language, and literacy barriers, availability and connectivity issues are also potential barriers to consider when developing these types of mHealth tools. Nevertheless, the strong attachment people have to mobile phones and the tendency to carry them everywhere open up opportunities for continuous symptom monitoring and connecting patients with providers outside of health care facilities.

### Limitations and Strengths

There are some limitations of this study. First, we did not perform a meta-analysis and we did not weigh the quality of evidence or study design against reported results. Second, only the English literature was included, and the sample size of the included studies varied substantially from 1 in a case report to 10,264 in a cohort study. Third, the diversity of study designs, objectives, and outcome measures made clear comparisons among studies difficult, and the quality of evidence was deemed to be variable. However, this is the first 25-year systematic review focusing on evidence collected from January 1999 to December 2023 regarding the impact of mHealth on CMP management adherence. The main strengths of this review are that we included a diverse array of study designs; assessed both self-management and clinical outcomes; and incorporated the nascent literature regarding mHealth feasibility, usability, and acceptability.

### Conclusions

mHealth is a potential high-impact tool to improve health outcomes among those with CMP through supporting adherence to personalized or tailored self-management programs for pain. Further evaluation of mHealth tools is needed, especially research that informs the cost-effectiveness of these tools. More innovation, optimization, and high-quality research in mHealth has the potential to transform the promise of mHealth technology into the reality of improved health care delivery and outcomes.

## Appendix

Multimedia Appendix 1 Search strategy.

Multimedia Appendix 2 Inclusion criteria for study selection and mHealth tools.

Multimedia Appendix 3 Eligible studies.

Multimedia Appendix 4 PRISMA (Preferred Reporting Items for Systematic Reviews and Meta-Analyses) flow for study selection.

Multimedia Appendix 5 Exluded studies with reasons.

Multimedia Appendix 6 Four types of mobile health tools.

Multimedia Appendix 7 Included studies investigating the impact of mobile health tools on adherence.

Multimedia Appendix 8 Includied studies investigating the impact of mobile health tools on clinical outcomes.

Multimedia Appendix 9 Included studies investigating the usability, feasibility, and acceptability of mobile health tools for patients with chronic musculoskeletal pain.

Multimedia Appendix 10 PRISMA 2020 checklist.

## Abbreviations

CLBP: chronic low back pain
CMP: chronic musculoskeletal pain
CNP: chronic neck pain
HCP: health care provider
ICD-11: International Classification of Diseases, 11th edition
MeSH: Medical Subject Headings
mHealth: mobile health
OA: osteoarthritis
PRISMA: Preferred Reporting Items for Systematic Reviews and Meta-Analyses
PROSPERO: International Prospective Register of Systematic Reviews
QoL: quality of life
RCT: randomized controlled trial
