# Supplementary material for: Impact of 25 Years of Mobile Health Tools for Pain Management in Patients With Chronic Musculoskeletal Pain: Systematic Review
Source: J Med Internet Res. 2024 Aug 16;26:e59358. doi: 10.2196/59358 (PMC11364951; doi:10.2196/59358)
Supplement: Multimedia Appendix 1 [file jmir_v26i1e59358_app1.docx]

**Multimedia Appendix 1. Search strategy.**

| Step #1 related to chronic musculoskeletal pain | Search (“cervical pain” OR “neck pain” OR “shoulder pain” OR “thoracic pain” OR “back pain” OR “low back pain” OR “joint pain” OR “arthralgia” OR “knee pain” OR “ankle pain” OR “limb pain” OR “osteoarthritis NOT structural” or “degenerative joint*”) |
| --- | --- |
| Step #2 related to  mHealth interventions | Search (mhealth OR "m-health" OR smartphone OR mobile* OR iphone OR ipad) |
| Step #5 related to  mHealth interventions | Search (app OR apps OR application*) |
| Step #4 related to study design | Search ("meta-analysis" or "systematic review" or "review") |
| Step #5 | Search (#1 AND #2 AND #3 NOT #4) |

PubMed

| ((((mhealth[Title/Abstract] OR "m-health"[Title/Abstract] OR smartphone[Title/Abstract] OR mobile*[Title/Abstract] OR iphone[Title/Abstract] OR ipad) (app[Title/Abstract] OR apps[Title/Abstract] OR application*[Title/Abstract])) AND (("cervical pain"[Title/Abstract] OR "neck pain"[Title/Abstract] OR "shoulder pain"[Title/Abstract] OR "thoracic pain"[Title/Abstract] OR "back pain"[Title/Abstract] OR "low back pain"[Title/Abstract] OR "joint pain"[Title/Abstract] OR "arthralgia"[Title/Abstract] OR "knee pain"[Title/Abstract] OR "ankle pain"[Title/Abstract] OR "limb pain"[Title/Abstract] OR "osteoarthritis"[Title/Abstract] OR "degenerative joint*"[Title/Abstract]))) AND ((app[Title/Abstract] OR apps[Title/Abstract] OR application*[Title/Abstract])) AND (1999:2024[pdat])) NOT ((("review"[Publication Type] OR "meta-analysis"[Publication Type])) OR (("review"[Title] OR "meta-analysis"[Title]))) Filters: English, from 1999 – 2023. |
| --- |

Cochrane CENTRAL

| #1 (“cervical pain” OR “neck pain” OR “shoulder pain” OR “thoracic pain” OR “back pain” OR “low back pain” OR “joint pain” OR “arthralgia” OR “knee pain” OR “ankle pain” OR “limb pain” OR “osteoarthritis NOT structural” or “degenerative joint*”)  #2 (mhealth OR "m-health" OR smartphone OR mobile* OR iphone OR ipad) (app OR apps OR application*)  #3 (app OR apps OR application*)  #4 (journal):pt  #5 English:la  #6 ((NOT PubMed)):an  #7 ((NOT Embase)):an  #8 #1 AND #2 AND #3  #9 #4 AND #5  #10 #8 AND #9  #11 #10 AND #6 AND #7 with Cochrane Library publication date Between Jan 1975 and Mar 2023, in Trials |
| --- |

MEDLINE

| 1 ("cervical pain" or "neck pain" or "shoulder pain" or "thoracic pain" or "back pain" or "low back pain" or "joint pain" or "arthralgia" or "knee pain" or "ankle pain" or "limb pain" or "osteoarthritis NOT structural" or "degenerative joint*").mp. (119980)  2 (mhealth or "m-health" or smartphone or mobile* or iphone or ipad).mp. (126085)  3 (app or apps or application*).mp. (1247978)  4 1 and 2 (637)  5 3 and 4 (180)  6 ("meta-analysis" or "systematic review" or "review").mp. (3658235)  7 5 not 6 (153)  8 limit 7 to (english language and yr="1999 - 2023") (149) |
| --- |

EMBASE

| 1 ("cervical pain" or "neck pain" or "shoulder pain" or "thoracic pain" or "back pain" or "low back pain" or "joint pain" or "arthralgia" or "knee pain" or "ankle pain" or "limb pain" or "osteoarthritis NOT structural" or "degenerative joint*").mp. (298126)  2 (mhealth or "m-health" or smartphone or mobile* or iphone or ipad).mp. (231944)  3 (app or apps or application*).mp. (1965272)  4 1 and 2 (1904)  5 3 and 4 (487)  6 ("meta-analysis" or "systematic review" or "review").mp. (4922661)  7 5 not 6 (399)  8 limit 7 to (english language and "remove medline records" and embase and yr="1999 - 2023" and (article or article in press)) (82) |
| --- |

Web of Science

| (((((TS=((“cervical pain” OR “neck pain” OR “shoulder pain” OR “thoracic pain” OR “back pain” OR “low back pain” OR “joint pain” OR “arthralgia” OR “knee pain” OR “ankle pain” OR “limb pain” OR “osteoarthritis NOT structural” or “degenerative joint*”))) AND TS=((mhealth OR "m-health" OR smartphone OR mobile* OR iphone OR ipad))) AND TS=((app OR apps OR application*) )) NOT ALL=(("meta-analysis" or "systematic review" or "review"))) AND DT=(Article)) AND DOP=(1999-01-01/2023-12-31). |
| --- |
