# Supplementary material for: Impact of 25 Years of Mobile Health Tools for Pain Management in Patients With Chronic Musculoskeletal Pain: Systematic Review
Source: J Med Internet Res. 2024 Aug 16;26:e59358. doi: 10.2196/59358 (PMC11364951; doi:10.2196/59358)
Supplement: Multimedia Appendix 2 [file jmir_v26i1e59358_app2.docx]

**Multimedia Appendix 2. Inclusion criteria**

| Study selection | 1. mHealth interventions aimed at improving CMP in studies were included even if the research did not address adherence specifically. 2. Usability, feasibility, and acceptability studies that focused on the design and development stages of mHealth interventions were included as a necessary precursor to future evaluation. 3. Studies that measured adherence included outcomes such as use of the mHealth tool for monitoring and reporting symptoms, compliance with medication regimens, and engagement in healthy behaviours. 4. Studies that focused on clinical outcomes of pain along with the functional ability or disability and QoL were included, as improved clinical outcomes are the eventual goal of improving adherence and often indicate adherence to CMP indirectly. |
| --- | --- |
|  |  |
| mHealth tools | mHealth include any mobile device or service, such as mobile phones, SMS, smartphones, personal digital assistants, and devices that work on wireless technology or Bluetooth-compatible devices. These devices and services allowed patients to monitor their health, access health information, and communicate with their health care provider without requiring a wired connection to the Internet. We included interventions delivered using a Web-based platform only if it was specified that the patient accessed the service via a mobile phone or other mobile device. It was required that patients be the primary users of the mHealth tools. |
|  |  |
