# Supplementary material for: Impact of 25 Years of Mobile Health Tools for Pain Management in Patients With Chronic Musculoskeletal Pain: Systematic Review
Source: J Med Internet Res. 2024 Aug 16;26:e59358. doi: 10.2196/59358 (PMC11364951; doi:10.2196/59358)
Supplement: Multimedia Appendix 3 [file jmir_v26i1e59358_app3.docx]

**Appendix 3. Eligible studies**

| Reference | Year | Country | Study design | mHealth type | Targeting population | Sample size | Follow-up duration | intervention group (N) | control group (N) | primary outcome | Secondary outcome | Other outcomes |
| --- | --- | --- | --- | --- | --- | --- | --- | --- | --- | --- | --- | --- |
| Øverås et al [20] | 2022 | Norway | Cohort | Mobile App | Patients with CLBP | 461 | 9 months | Intervention arm: the SELFBACK system in addition to usual care (n=232) | Control arm: usual care only (n=229) | LBP-related disability (Roland Morris Disability Questionnair, RMDQ) | Stress/depression/illness perception/self-efficacy/general health/quality of life/physical activity/global perceived effect |  |
| Abadiyan et al [21] | 2021 | Iran | RCT | Mobile App | Patients with nonspecific neck pain | 60 | 8 weeks | Group 1 (8-week global postural reeducation [GPR], a smartphone app, n=20) | Group 2 (GPR alone, n = 20); Group 3 (the control group, n=20) | Pain during the last 24 h | Disability, quality of life, endurance, and posture. |  |
| Ackerman et al [22] | 2017 | Australia | Questi-onnaire | Mobile App | Younger people with OA | 147 | NA | NA | NA | Disease-related education and support services, as well as perceived usefulness and accessibility of delivery modes including group-based programs, online resources, telephone helplines, mailed information, social media, and mobile applications | Quality of Life, Kessler Psychological Distress, and Western Ontario and McMaster Universities Osteoarthritis Index (WOMAC) |  |
| Alasfour et al [23] | 2022 | Saudi Arabia | RCT | Mobile App | Women with KOA | 40 | 6 weeks | The app group receiving home exercise programs (HEPs) using an Arabic smartphone application called “My Dear Knee” (n = 20) | The paper group receiving HEPs as hand-outs (n = 20) | Self-reported exercise adherence | Arabic numeric pain rating scale, Five-times sit-to-stand test |  |
| Amorim et al [24] | 2019 | Australia | RCT | Web-based mobile App and monitor | Patients with chronic low back pain (CLBP) | 68 | 6 months | A physical activity information booklet, plus one face-to-face and 12 telephone-based health coaching sessions (n=34) | Receiving the physical activity information booklet and advice to stay active (n=34) | Care-seeking, pain levels and activity limitation |  | Recruitm-ent rate, intervention compliance, data completen-ess, and participant satisfaction |
| Anan et al [25] | 2021 | Japan | RCT | Mobile App | Workers either with neck /shoulder stiffness /pain or low back pain or both | 94 | 12 weeks | An exercise-based artificial intelligence (AI)–assisted interactive health promotion system that operates through a mobile messaging app (the AI-assisted health program) for 12 weeks (n=48) | Usual care routines (n=46) | Subjective severity of the neck and shoulder pain/stiffness and low back pain | Subjective assessment of whether there was an improvement. |  |
| Arensman et al [26] | 2022 | Netherlands | Qualita-tive study | Mobile App | Patients with non-Specific CLBP | 9 | NA | NA | NA | Patient perspectives on the acceptability, satisfaction, and performance of a smartphone app to support home-based exercise following recommendations from a physical therapist. |  |  |
| Arfaei Chitkar et al [27] | 2021 | Iran | RCT | Mobile App | Women KOA | 64 | 2 months | Intervention group received mobile-app-based instruction coupled with routine cares (n=32) | Comparison group just received the routine cares (n=32) | Pain, stiffness, and function of joints by WOMAC | SF-36 |  |
| Östlind et al [28] | 2022 | Sweden | Qualita-tive study | Mobile App | individuals of working age with hip OA and KOA from a cluster-randomized controlled trial | 18 | NA | The intervention in the C-RCT comprised of 12-weeks use of a WAT with a mobile application to monitor PA. | NA | Experiences and perceptions |  |  |
| Bailey et al [29] | 2020 | United States | Cohort | Mobile App | Patients with chronic musculoskeletal pain (CMP) | 10,264 | 12 weeks | A 12-week remotedigi-tal care program (DCP) available through a mobile app incorporating education, sensor-guided exercise therapy (ET), and behavioral health support with 1-on-1 remote health coaching | NA | Pain measured by the visual analog scale (VAS) | Engagement levels, program completion, program satisfaction, condition-specific pain measures, depression, anxiety, and work productivity. |  |
| Barber et al [30] | 2019 | Canada | Qualita-tive study | Mobile App | KOA patients and family physicians | 9 | NA | NA | NA | Perspectives of family physicians and patients on KOA, its treatment/management and the use of a mobile health application (app) to help patients self-manage their KOA |  |  |
| Bechler et al [31] | 2021 | Germany | Cohort | Mobile App | Patients with end-stage OA | 93 | NA | Intraoperative software app-based anteversion measurements (n=93) | Standard postoperative AP-pelvis radiographs (n=93) | Postoperative dislocation |  |  |
| Bellamy et al [32] | 2010 | Australia | Pilot study | Mobile App | KOA and Hip OA patients | 12 | Mean 4.8 minutes | A mobile phone technology for repeated independent remote data capture using the mobile phone-based m-WOMAC® NRS 3.1 Index. (n=12) | NA | Experience with independent use of the m-WOMAC® application. |  |  |
| Bellamy et al [33] | 2011 | Australia | Cohort | Mobile App | Patients with OA undergoing hip or knee joint replacement | 62 | 3-4 months | Osteoarthritis Index delivered by mobile phone (m-WOMAC) | NA | Validity, reliability, and responsiveness |  |  |
| Beresford et al [34] | 2022 | United States | Cohort | Mobile App | Musculoskeletal condition such as low back pain, neck pain, arthritis, sprains, strains, or similar overuse injuries that would benefit from PT or presented for postoperative rehabilitation | 814 | Average 44 days | Digital PT | NA | Pain levels, level of functional impairment |  |  |
| Biebl et al [35] | 2020 | Germany | Questi-onnaire | Mobile App | Patients with KOA and hip OA | 127 | NA | NA | NA | Attitudes of medical professionals toward app-based therapy |  |  |
| Biebl et al [36] | 2021 | Germany | Cohort | Mobile App | KOA and Hip OA | 24 | NA | Motion Coach app feedback (n=24) | Physiotherapists' feedback (n=24) | Overall agreement between physiotherapists’ and Motion Coach’ evaluations during exercise execution. |  |  |
| Chhabra et al [37] | 2018 | India | RCT | Mobile App | Patients with CLBP | 93 | 12 weeks | App group receiving S-nap care, in addition to the written prescription (n = 45) | The Conventional group receiving a written prescription from the Physician, containing a list of prescribed medicines and dosages, and stating the recommended level of physical activity (including home exercises) (n = 48) | Pain and disability for both the groups at baseline and at the end of 12 weeks | Daily physical activity (distance measured through an activity tracker built within the app) and progress in symptoms through the CSS (Current Symptom Score). |  |
| Colomina et al [38] | 2021 | Spain | Cohort | Mobile app and monitor | Patients with OA undergoing primary hip or knee arthroplasty | 69 | 3 months | mHealth-enabled Integrated care (IC) arm with a self-management app and a Fitbit Flex 2 digital activity tracker (n=39) | The usual care (UC) arm (n=30) | Intervention effectiveness (SF-12) | Use of health care resources after 6 months | Cost-effectivene-ss, based on the improvem-ent in QoL relative to costs, assessed by means of the incremental cost-effectivene-ss ratio (ICER). |
| Correia et al [39] | 2019 | Portugal | Pilot study | Mobile app and monitor | Patients admitted to hospital for primary TKA | 59 | 6 months | A 8w digital intervention uses a motion tracker allowing 3D movement quantification, a mobile app and a Web portal (n=30) | Conventional face-to-face sessions (n=29) | Timed Up and Go score, and secondary outcomes were the Knee Osteoarthritis Outcome Scale (KOOS) score and knee range of motion. |  |  |
| Crawford et al [40] | 2021 | United States | RCT | Mobile App | Patients undergoing primary TKA and partial knee arthroplasty PKA | 452 | 4 weeks | The treatment group were provided a smartwatch and smartphone application (n = 208) | The control group received the respective institution’s standard of care with formal physiotherapy (n = 244) | 90-day knee range of movement, EuroQoL five-dimension five-level score, Knee Injury and Osteoarthritis Outcome Score for Joint Replacement (KOOS JR) score, 30-day single leg stance (SLS) time, Time up and Go (TUG) time, and need for manipulation under anaesthesia (MUA) |  |  |
| Crawford et al [41] | 2021 | United States | RCT | Mobile App | Patients undergo a unilateral primary THA | 265 | 90 days | Treatment group were provided with a smartwatch and smartphone application(n=167) | Control group received the institution’s standard of care(n=198) | Physiotherapy (PT) use, THA complications, readmissions, emergency department/urgent care visits, and physician office visit |  | Satisfaction scores for the procedure and the smartphone-based care system group were also recorded. |
| Dasa et al [42] | 2022 | United States | RCT | Mobile App | Patients with KOA | 29 | 14 weeks | “Active NMES”: home-based NMES (two 20-minute daily sessions, 5 d/wk) with either the original device (n=21) | “Sham NMES”: home-based NMES (two 20-minute daily sessions, 5 d/wk) with a low-voltage version (n=8) | Patient-reported outcome measures included VAS, WOMAC,36 and a 7-point Patient Global Impression of Change (PGIC) |  | Patient's satisfaction |
| deBritoMacedo et al [43] | 2020 | Brazil | Cross-section-al Study | Mobile App | Patients with CLBP | 40 | 1 day | NA | NA | Concurrent validity by the Intraclass Correlation Coefficients (ICC) and intrarater reliability | The mobile application may be considered a valid and reliable tool to assess thoracolumbar ROM for both asymptomatic and chronic low back pain subjects. |  |
| Delgado et al [44] | 2023 | United States | Cohort | Mobile App | Patients with CMP including pain sites: “ankle,” “foot,” “hand,” “hip,” “knee,” “lower back,” “neck,” “shoulder,” “upper back,” and “wrist”. | 3109 | 8-12 weeks | A mHealth-guided exercise therapy program (n=3109) | NA | Pain intensity was assessed via the app at baseline and at the beginning of each session using a numeric rating scale (NRS) | Quality of Life, Work Life, and Program Engagement |  |
| Guetin et al [45] | 2016 | France | Cohort | Mobile App | Patients with different chronic pain conditions | 53 | 20 minutes | The Music Care application is a receptive music intervention, allowing the patient to listen to a standardized musical sequence of 20 min in length (n=53) | NA | Pain intensity on a visual analogue scale (VAS) integrated within the application, with anchors at 0 (no pain) and 10 (most intense pain) | Anxiety and satisfaction with the intervention. the feasibility and general usability of the application |  |
| Han et al [46] | 2022 | United States | Cohort | Mobile app and monitor | People with chronic pain | 312 | 12 months | Group 1: a multidisciplinary pain program that included WHT use (n=105) | Group 2: the same program but without WHT (n=146); Group 3: patients receiving medical pain management without WHT (n=161) | Patient Health Questionnaire-9 (PHQ-9), numeric rating scale (NRS), Oswestry Disability Index (ODI) Neck, ODI Back, and morphine milligram equivalents (MME) | Opioid use and morphine milligram equivalents (MME) |  |
| Hardt et al [47] | 2018 | Germany | RCT | Mobile App | Patients awaiting primary TKA for treatment-ng primary end-stage OA | 60 | Mean 7 days | The training group postoperatively performed an app-based feedback-controlled active muscle training programme multiple times daily (n=33) | The control group follow standardized identical postoperative protocol and identical pain management (n=27) | Active range of motion (ROM) measured using a goniometer (Medigauge® Digital Protractor Goniometer for Medical applications, Taylor Tool works LLC, Columbia, USA) and rounded to the next integer and pain at rest and in motion measured with the numeric rating scale (NRS) as 24-h summary, and (3) maximum knee strength as recorded by the GenuSport application | Passive ROM, three assessment tests (timed “Up and Go”, 10-m Walk Test, 30-s Chair Stand Test), and two scores [Knee Injury and Osteoarthritis Outcome Score (KOOS), Knee Society Score (KSS)] to evaluate function and patient reported outcomes |  |
| Hartmann et al [48] | 2023 | Germany | Cohort | Mobile App | Patients with CLBP | 51 | 8 weeks | The intervention group with 8-week app-based exercise program (n=35) | A so-called rehabilitation sports group served as the control group (16) | Pain score using the NRS | Pain-related impairment in daily living, measured with the oswestry disability index (ODI) |  |
| Hoogland et al [49] | 2019 | Netherlands | Cohort | Mobile app and monitor | OA patients undergoing total hip arthroplasty (THA) | 30 | 6 months | A 12w home-based rehabilitation program driven by a tablet app and mobility monitoring (n=30) | NA | Feasibility and Patient Experience of a Home-Based Rehabilitation Program Driven by a Tablet App and Mobility Monitoring |  | Adherence |
| Huber et al [50] | 2017 | Germany | Cohort | Mobile App | Patients with unspecific low back pain | 180 | 12 weeks | The Kaia app | NA | Pain levels measured by NRS |  |  |
| Itoh et al [51] | 2022 | Japan | RCT | Mobile App | Patients with CLBP | 99 | 12 weeks | Receiving education and exercise therapy using a mobile messaging app (n=48) | Receiving usual medical care with pharmacological treatment (n=51) | Pain intensity, work productivity, | Quality of life, fear of movement, and depression. |  |
| Kerckhove et al [52] | 2022 | France | Pilot study | Mobile App | Patients with chronic pain | 105 | 3 months | Regular self-monitoring using an mHealth app (n=105) | NA | Feasibility and acceptability | Satisfaction surveys from both patients and physicians. |  |
| Kravitz et al [53] | 2018 | United States | RCT | Mobile App | Patients with CMP | 215 | 1 month | The 6-week n-of-1 intervention supported by a mobile health (mHealth) app (n=105) | The control group (n=107) | Change in the PROMIS (Patient-Reported Outcomes Measurement Information System) pain-related interference 8-item short-form scale (full scale range, 41-78) from baseline to 6 months. | Patient-reported pain intensity, overall health, analgesic adherence, trust in clinician, satisfaction with care, medication-related shared decision making, | Participant engageme-nt and experience for the n-of-1 group only. |
| Krkoska et al [54] | 2023 | Czech Republic | Pilot study | Mobile App | Patients with non-Specific CLBP | 27 | 18 weeks | Mobile app use | NA | Adherence and effectiveness |  | Patient-oriented and functional outcomes |
| Kurtz et al [55] | 2022 | Korea | Questi-onnaire | Mobile App | Patients with hip OA and/or total hip arthroplasty (THA) | 97 | NA | NA | NA | Willingness and adherence |  |  |
| Lambert et al [56] | 2017 | Australia | RCT | Mobile App | Patients with upper or lower limb musculoskeletal conditions | 80 | 4 weeks | The intervention group received their home exercise programs on an app linked to the freely available website and supplemen-tary phone calls and motivational text messages (n = 40) | The control group received their home exercise programs as a paper handout (n = 40) | Self-reported exercise adherence. | Functional performance, disability, patient satisfaction, perceptions of treatment effectiveness, and different aspects of adherence. |  |
| Lebleu et al [57] | 2023 | Belgium | Cohort | Mobile App | Patients with degenerative knee pain who utilized digital rehabilitation following TKA | 127 | 1 year | The home-based digital intervention of exercise and education (n=127) | NA | The drop-out rate, complications and readmissions, PROMS, and satisfaction |  |  |
| Lee et al [58] | 2017 | Korea | Cohort | Mobile App | Office Workers with Neck Pain | 23 | 8 weeks | A self-classification algorithm implemented as a smartphone application, and conducted corresponding exercise programs (n=23) | NA | The visual analog scale (VAS), Neck Disability Index (NDI), | Medical Outcomes Study 36-Item Short-Form Health Survey (SF-36), Fear-Avoidance Beliefs Questionnaire (FABQ), and cervical ROM | Applicability outcomes included patient satisfaction and adherence |
| Lee et al [59] | 2017 | Korea | Pilot study | Mobile App | Office workers with chronic neck pain and functional disability | 20 | 8 weeks | The app-based exercise group conducted neck exercise through the mobile app in the workplace environment for at least 10–15 min/day, 2 days/week for 8 weeks (n = 11) | The control group received a brochure showing how to correct their posture themselves during the same period (n = 9) | Visual Analog Scale (VAS) for pain intensity; Neck Disability Index (NDI), | Functional disability; level of exercise adherence; maximal voluntary flexion/extension strength (MVFS/MVES); 36-Item Short-Form Healthy Survey (SF-36), for quality of life; and Fear-Avoidance Belief Questionnaire (FABQ). |  |
| Lewkowicz et al [60] | 2022 | United States | Cost-Effecti-veness | Mobile App | Patients with nonspecific low back pain | NA | 3 years | NA | NA | Incremental cost and quality-adjusted life years (QALYs) |  |  |
| Lin et al [61] | 2019 | United States | Pilot study | Mobile App | Patients with CLBP | 18 | 4 weeks | A 4-week auricular point acupressure intervention | NA | Pain intensity, pain interference with daily activity, sleep quality score, and medication usage | Feasibility and usability | System Usability Scale and adherence |
| Lo et al [62] | 2018 | China | Questi-onnaire | Mobile App | Chronic Neck and Back Pain | 161 | NA | NA | NA | Increase time spent on therapeutic exercise, affect pain level (assessed by the 0-10 Numerical Pain Rating Scale), and reduce the need for other interventions. |  |  |
| Marcuzzi et al [63] | 2023 | Norway | RCT | Mobile App | Patients with neck and/or low back pain | 294 | 6 months | The App group: Receiving app-based individually tailored self-management support in addition to usual care (n=99) | The e-Help group: web-based nontailored self-management support in addition to usual care (n=98); usual care group: usual care alone (n=97) | Change in musculoskeletal health measured by the Musculoskeletal Health Questionnaire (MSK-HQ) at 3 months | Change in musculoskeletal health measured by the MSK-HQ at 6 weeks and 6 months and pain-related disability, pain intensity, pain-related cognition, and health-related quality of life at 6 weeks, 3 months, and 6 months. |  |
| Mbada et al [64] | 2019 | Nigeria | Pilot study | Mobile App | Patients with CLBP | 54 | 8 weeks | Telerehabilitation-based McKenzie (TBMT)group performed in the home with the assistance of a mobile phone app (n=32) | Clinic-Based McKenzie Therapy (CBMT) group receiving the McKenzie extension protocol (n=24) | Pain intensity, back extensors muscles’ endurance, | Activity limitation, participation restriction, general health status | Cost-utility |
| Neumann-Langen et al [65] | 2023 | Germany | Pilot study | Mobile app and wearable sensors | Patients undergoing primary total knee arthroplasty (TKA) | 98 | 6 weeks | Mobile app and wearable sensors use during the postoperative course of primary total knee arthroplasty (n=98) | NA | No specific primary endpoint is defined due to the prospective nature of this study |  |  |
| Nordstoga et al [66] | 2020 | Norway | Cross-section-al Study | Mobile App | Patients with non-specific CLBP | 16 | NA | NA | NA | Usability and acceptability |  |  |
| Al Saadawy et al [67] | 2021 | Egypt | Cross-section-al study | Mobile App | Patients with bilateral OA and asymptomatic controls | 32 | NA | NA | NA | Joint position sense (JPS) |  |  |
| Pach et al [68] | 2022 | Germany | RCT | Mobile App | Patients with chronic neck pain (CNP) | 220 | 6 months | To practice app-based relaxation exercises (n=110) | Usual care and app for data entry only (n=110) | Mean neck pain intensity during the first 3 months | Neck pain based on weekly measurements, pain acceptance, neck pain–related stress, sick-leave days, pain medication intake, and adherence |  |
| Park et al [69] | 2023 | Korea | RCT | Mobile App | Patients with CLBP | 100 | NA | Digital application physical therapy (DPT) group: two days of Dr AI in addition to an initial in-person meeting with a physical therapist (n=50) | Conventional physical therapy (CPT) group (n=50) | Back pain intensity, limited functional ability, lower extremity weakness, radicular symptoms, limited range of motion (ROM), functional movement, quality of life, cost-effectiveness | Perceived transmission risk of COVID-19 and satisfaction results in 100 participants with low back pain (LBP) |  |
| Pelle et al [70] | 2020 | Netherlands | RCT | Mobile App | Patients with KOA and/or Hip OA | 427 | 6 months | The dr. Bart group used the dr. Bart app (n=214) | The usual care group received no active treatment (n=213) | The number of secondary health care consultations |  |  |
| Pelle et al [71] | 2021 | Netherlands | Cohort | Mobile App | People withKOA and/or hip OA | 214 | 6 months | Intervention group of an RCT examining the effectiveness of the dr. Bart app (n = 214) | NA | The effectiveness of the dr. Bart app | The usability of the app measured using the System Usability Scale questionnaire (SUS) | Association between the intensity of use of the app and health care utilisation (i.e., consultations in primary or secondary health care) and clinical outcomes (i.e., self-management behaviour, physical activity, health-related quality of life, illness perceptions, symptoms, pain, activities of daily living) |
| Peterson et al [72] | 2018 | United States | Case series | Mobile App | Patients with CLBP | 3 | 12 months | NA | NA | Functional status was measured using the ODI, Low self-efficacy, Pain intensity, |  | Adherence and feasibility |
| Pourahmadi et al [73] | 2018 | Iran | Cross-section-al study | Mobile App | Patients with non-specific neck pain | 40 | NA | NA | NA | Active craniocervical ROM (ACCROM) including flexion, extension, lateral flexion, and rotation. |  |  |
| Pourahmadi et al [74] | 2021 | Iran | Cross-section-al study | Mobile App | Patients with nonspecific chronic low back pain (CLBP) | 15 | NA | NA | NA | Reliability and validity measuring active lumbar flexion-extension range of motion (ROM) |  |  |
| Pourahmadi et al [74] | 2021 | Iran | Cross-section-al study | Mobile App | Patients with nonspecific CNLBP | 15 | NA | NA | NA | Active lumbar spine maximum flexion and maximum extension range of motion |  |  |
| Rabbi et al [75] | 2018 | United States | Pilot study | Mobile App | Patients with CLBP | 10 | 5 weeks | Intervention group received generic recommendations from an expert for 2 weeks and “MyBehaviorCBP” recommendations for next 2 weeks (n=10) | Control group received generic recommendations from an expert for 2 weeks (n=10) | Feasibility of “MyBehaviorCBP”, which was measured by 3 factors: use, acceptability, and early efficacy |  |  |
| Rafiq et al [76] | 2021 | Malaysia | RCT | Mobile App | Overweight and obese patients with KOA | 114 | 3 months | Rehabilitation group with mHealth (RGw-mHealth) to receive LLRP + instructions of daily care (IDC) combined with mHealth intervention (n=38) | Rehabilitation group without mHealth (RGwo-mHealth) to receive LLRP + IDC intervention (n=38); control group (CG) to receive IDC intervention (n=38) | Knee pain symptoms assessed using the Western Ontario and McMaster Universities Osteoarthritis Index (WOMAC) | Mobility, functional activity, and ADL. |  |
| Ramkumar et al [77] | 2019 | United States | Pilot study | Mobile app and wearable sensors | OA patients undergoing primary TKA | 25 | 3 months | Wearable knee sleeve paired mobile app use | NA | Validation was determined by acquisition of continuous data and patient tolerance at semistructur-ed interviews 3 months after operation. |  |  |
| Ramos et al [78] | 2019 | Brazil | Cross-section-al study | Mobile App | Patients with shoulder pain | 25 | 1 week | NA | NA | Active joint repositioning tests of shoulder flexion and scaption and elbow flexion |  |  |
| Rodriguez Sanchez-Laulhe et al [79] | 2023 | Spain | RCT | Mobile App | Patients with unilateral or bilateral hand OA | 74 | 12 weeks | A home exercise program (4 times a week) over 12 weeks, delivered with the CareHand mobile app (n=66) | Usual care using a paper sheet that included pictures and explanations of exercises and dosage (n=57) | Self-reported hand physical function at 3- and 6-months postintervention | Self-reported measures of hand overall status, pain intensity and morning stiffness, and upper limb function |  |
| Rouzaud Laborde et al [80] | 2021 | United States | Pilot study | Mobile app and wearable sensors | Patients with KOA | 28 | 2 weeks | Mobility monitoring (ROAMM) mobile app designed for smartwatches | NA | Satisfaction, usability, and compliance |  |  |
| Rughani et al [81] | 2023 | Norway | Cohort | Mobile App | Patients with CLBP | 461 | 9 months | Usual care plus selfBACK app (n = 232) | Usual care (n = 229) | LBP-related disability (Roland–Morris Disability Questionnaire, RMDQ) over 9 months | Global perceived effect (GPE)/pain self-efficacy (PSEQ)/satisfaction/app engagement. Baseline depressive symptoms were measured using the patient health questionnaire (PHQ-8) and stress with the perceived stress scale (PSS). |  |
| Sandal et al [82] | 2021 | Denmark | RCT | Mobile App | Patients with CLBP | 461 | 9 months | The intervention group to receive the selfBACK self-management support system in addition to usual care (n=232) | The control group instructed to manage their LBP according to the advice or treatment offered by their clinician (n=229) | Mean difference in RMDQ scores between the intervention group and control group at 3 months. | Average and worst LBP intensity levels, ability to cope as, fear-avoidance belief, cognitive and emotional representations of illness, health-related quality of life, physical activity level, and overall improvement |  |
| Sax et al [83] | 2022 | United States | RCT | Mobile App | Patients with KOA | 156 | 12 weeks | A home-based NMES therapy (n=106) | A modified low-voltage NMES therapy (sham therapy) (n=50) | Percentage change from baseline (PCFB) in the Visual Analog Scale (VAS) pain | Knee pain measured by VAS, WOMAC Index, KOOS Joint Replacement, and isometric quadriceps strength test |  |
| Selter et al [84] | 2018 | United States | Cohort | Mobile App | patients with CLBP | 93 | 3 months | mHealth-based 3-month physical therapy program (Limbr) and received a mobile phone app suite free of charge to monitor and manage their CLBP. | NA | 1. Patient engagement using three outcome variables: (1) the frequency of interactions across the visual self-reports, (2) a binary outcome representing at least one viewing of the physical therapy videos versus none watched, and (3) the frequency of messages to the health coach. 2. Patient-Perceived Utility of Limbr by web-based survey |  |  |
| Shah et al [85] | 2023 | United States | Qualita-tive study | Mobile App | Patients with KOA | 36 | NA | NA | NA | Perceptions of using smartphones | Rating and perceptions of specific app features |  |
| Shebib et al [86] | 2019 | United States | RCT | Mobile App | Patients with non-specific CLBP | 117 | 12 weeks | A 12-week digital care program (DCP) (n=113) | Three digital education articles only(n=64) | Oswestry Disability Index, Korff Pain and Korff Disability |  | Understanding of LBP and reduction in back surgery interest |
| Shewchuk et al [87] | 2021 | Canada | Qualita-tive study | Mobile App | Patients with early KOA and health care providers (HCPs) | 25 | 6 weeks | NA | NA | Qualitative and Quantitative Evaluations for KOA and HCPs |  |  |
| Sitges et al [88] | 2022 | Spain | RCT | Mobile App | Patients with nonspecific chronic low back pain (CLBP) | 59 | 4 weeks | Self-managed intervention group with moble app (n=23) | Face-to-face intervention group, they met with the supervisor (n=27) | Electroencephalographic activity (at rest and during a modified version of the Eriksen flanker task) and heart rate variability (at rest), PPTs, and pressure pain intensity ratings | Pain, disability, psychological functioning (mood, anxiety, kinesiophobia, pain catastrophizing, and fear-avoidance beliefs), and cognitive performance (percentage of hits and reaction times) |  |
| Skrepnik et al [89] | 2017 | United States | RCT | Mobile app and monitor | KOA patients treated with hylan G-F 20 | 211 | 90 days | Group A: Jawbone and OA GO with visible feedback (n=107) | Group B: Jawbone only with no visible feedback (n=104) | Mean change from baseline to day 90 in mobility as measured by steps per day | Mean percentage change from baseline in steps per day at each assessment visit (average of a 7-day period), and at day 90, mean percentage change from baseline in the 6-minute walk test (distance and pain assessed by the NPRS), patient and physician satisfaction with treatment, percentage change in Patient Activation Measure (PAM)-13 questionnaire score [30], percentage change in sleep captured by the wearable activity monitor (light, sound, and duration of sleep), and Visual Analog Mood Scale (VAMS) assessment. | Treatment-emergent adverse events (TEAEs) were also assessed. |
| Slater et al [90] | 2016 | Australia | Qualita-tive study | Multiple digital technologies | Young people persistent musculoskeletal pain including non-specific conditions (eg, low back pain) and specific conditions (eg, juvenile idiopathic arthritis and other systemic arthritides), with/without pre-existing or current diagnosed mental health conditions. | 23 | NA | NA | NA | NA |  |  |
| Slater et al [91] | 2020 | Australia | Cohort | Mobile App | Young Australians with musculoskeletal pain. | 15 | 3 months | Use of Digital Technologi-es (painHEALTH website and the iCanCope with Pain app) (n=15) | NA | Acceptability and need for adaptation of extant digital technologies |  |  |
| Støme et al [92] | 2019 | Norway | Pilot study | Mobile App | OA patients | 12 | 12 weeks | A 12-week mixed-mode goal achievement plan with digital support based on preset goals, self-monitoring and individual feedback (n=12) | NA | Utility and usability were assessed via 10 weekly questions and adherence by fulfilment of predetermined tasks. | Acceptability was measured as the perceived degree of goal achievement using a validated habit questionnaire scaled from 0 to 100. |  |
| Suso-Ribera et al [93] | 2023 | Spain | Cohort | Mobile App | Adults with heterogenous chronic pain | 38 | 1 month | Pain Monitor App (n=38) | NA | Validity, Reliability, Feasibility, and Usefulness |  |  |
| Suso-Ribera et al [94] | 2020 | Spain | RCT | Mobile App | Patients with CLBP | 132 | 1 month | The daily ecological momentary assessment using the Pain Monitor app with (n = 43) and without alarms (n = 45) | A usual monitoring method according to the usual practice at the pain clinic and pain guidelines (n=44) | Pain severity and side effects of the medication | Fatigue, pain interference and mood states, namely depression, anxiety and anger |  |
| Svendsen et al [95] | 2022 | Denmark | Qualita-tive study | Mobile App | Patients with CLBP | 26 | Mean 42 minutes | NA | NA | Factors facilitating and limiting the implementation of selfBACK emerged |  |  |
| Teepe et al [96] | 2023 | Germany | Cohort | Mobile App | Patients with unspecific and degenerative musculoskeletal pain | 3629 | 12 weeks | The app automatically composes an individualized set of 4 exercises from a repository of 120 different exercises. | NA | Self-reported pain score, which was assessed with a verbal numerical rating scale | Self-reported function scores |  |
| Thiengwittayaporn et al [97] | 2023 | Thailand | RCT | Mobile App | Patients with KOA | 82 | 4 weeks | The mobile application group (M-group, 44) | The handout group (H-group, 45) | Patient’s ability to correctly perform the three prescribed exercises | Clinical outcomes in terms of ROM, KOOS categories (symptoms, pain, activities of daily living, sports and recreation activities, and quality of life), and KSS categories (objective knee score, satisfaction, expectation, and functional activity) |  |
| Thongtipmak et al [98] | 2020 | Thailand | RCT | Mobile App | Patients with non-specific neck pain | 100 | 20 minutes | The treatment group cond-ucted the exercise program via smartphone application for 15–20 minutes (n = 50) | The control group rested for 20 minutes (n = 50) | Pain intensity, muscle tension, pressure pain threshold (PPT), and cervical range of motion (CROM) |  | Quantitative and qualitative feedback by an acceptability survey and open-ended questionnaires. |
| Toelle et al [99] | 2019 | Germany | RCT | Mobile App | Patients with non-specific low back pain | 101 | 3 months | The Kaia App (n=53) | Control treatment consisted of six individual physiotherapy sessions over 6 weeks and high-quality online education (n=48) | Pain symptoms, pain intensity | Functional ability and wellbeing (Hannover Functional Ability Questionnaire HFAQ), Graded Chronic Pain Scale (Graded Chronic Pain Scale GCPS), response rate | Concomitant pain medication, Kaia App activity, adherence |
| Tripuraneni et al [100] | 2021 | Mexico | RCT | Mobile app and wearable sensors | Patients undergoing primary total knee arthroplasty (TKA) | 337 | 8 weeks | Study group with a 2-week preoperative exercise regimen followed by a 6-week postoperative exercise regimen (n=153) | Control group with formal physical therapy [PT]) (n=184) | Patient-reported outcome measures (PROMs) of knee injury and osteoarthritis outcome scores, joint replacement (KOOS, JR), and EuroQol five-dimension five-level (EQ-5D-5L) along with range of motion (ROM) | Manipulation rates | Compliance |
| Vad et al [101] | 2022 | United States | Cohort | Mobile App | Patients with axial CLBP | 75 | 3 months | The BackRx app | NA | Back pain evaluated using the visual analog scale (VAS) for pain | Patient's functionality, the weekly pain medication intake | Patients’ adherence to the app, and the patients´ satisfaction rate. |
| VanWye et al [102] | 2018 | United States | Case report | Mobile App | Patinets with knee pain 24 months status-post triple arthrodesis following a work-related crush injury. | 1 | NA | NA | NA |  |  |  |
| Weise et al [103] | 2022 | Germany | RCT | Mobile App | Patients with unspecific and degenerative back pain | 213 | 12 weeks | The interventional group was provided access to the digital therapeutic on their mobile device (n=108) | The control group were assigned to receive physical therapy from a certified physiotherapist of their choice (n=105) | Self-reported pain intensity | Total pain scores and their changes during the study |  |
| Yamamoto et al [104] | 2022 | Japan | Pilot study | Mobile App | Patients with knee osteoarthritis (KOA) | 20 | 12 weeks | Participants performed the exercises using the application for 12 weeks | NA | Adherence rates (the total number of exercise days/84 × 100%) and the number of special ideogrammatic icons on the home screen calendar. | Satisfaction, Japanese knee osteoarthritis measure (JKOM) score, short physical performance battery (SPPB) score, knee extension muscle strength (Nm/kg) and short test battery (STB) for locomotive syndrome (LS) |  |
| Yang et al [105] | 2019 | China | RCT | Mobile App | Patients with CLBP | 8 | 4 weeks | SM + PT group: received self-management program through the use of an APP (n=5) | Control group received physiotherapy only (n=3) | Pain visual Analog Scale (VAS), Pain Self-Efficacy Questionnaire (PSEQ), Roland Morris Disability Questionnaire (RMDQ), and SF36. |  |  |
| Yeh et al [106] | 2022 | United States | Pilot study | Mobile App | Patients with CMP | 37 | 1 month | Self-guided auricular point acupressure smartphone app (mAPA) instructed virtually on APA protocol in an approximately 15 min session (n = 14) | The in-person mAPA group received in-person APA training at the recruitment site for a similar timeframe (n = 12); the waitlisted education-enhanced control group (n = 11) | Physical function and pain intensity |  | Adherence, satisfaction, Qualitative Findings |
| Zhuo et al [107] | 2021 | Canada | Cross-section-al study | Mobile App | Patients with nonspecific low back pain | 17 | 12 weeks | NA | NA | Pain (Numerical Rating Scale), function (Patient-Specific Functional Scale), disability (Roland Morris Disability Questionnaire), health-related quality of life (EQ-5D-5L), and physical activity (activity tracker and modified IPAQ) | Feasibility and convergent validity |  |
