# Supplementary material for: Impact of 25 Years of Mobile Health Tools for Pain Management in Patients With Chronic Musculoskeletal Pain: Systematic Review
Source: J Med Internet Res. 2024 Aug 16;26:e59358. doi: 10.2196/59358 (PMC11364951; doi:10.2196/59358)
Supplement: Multimedia Appendix 4 [file jmir_v26i1e59358_app4.docx]

**Multimedia Appendix 4. PRISMA diagram for study selection process.**
