# Supplementary material for: Impact of 25 Years of Mobile Health Tools for Pain Management in Patients With Chronic Musculoskeletal Pain: Systematic Review
Source: J Med Internet Res. 2024 Aug 16;26:e59358. doi: 10.2196/59358 (PMC11364951; doi:10.2196/59358)
Supplement: Multimedia Appendix 5 [file jmir_v26i1e59358_app5.docx]

**Excluded studies with exclusion reasons.**

| Study | Published Year | Accession Number | DOI | PMID | Exclusion reason |
| --- | --- | --- | --- | --- | --- |
| Yeh 2022 | 2022 |  | 10.1016/j.pmn.2021.11.007 | 34961729 | Exclusion reason: Wrong intervention; |
| Villatoro-Luque 2023 | 2023 |  | 10.1177/1357633X231195091 | 37649362 | Exclusion reason: No full text; |
| Irvine 2015 | 2015 | ClinicalTrials.gov/NCT01950091 | 10.2196/jmir.3130 | 25565416 | Exclusion reason: Wrong intervention; |
| Kurtz 2023 | 2023 |  | 10.1055/s-0042-1755378 | 36049771 | Exclusion reason: No full text; |
| Reed 2022 | 2022 |  | 10.1186/s12891-022-05376-9 | 35534813 | Exclusion reason: Wrong patient population; |
| Zheng 2022 | 2022 |  | 10.1186/s13063-022-06116-z | 35241140 | Exclusion reason: Wrong comparator; |
| Arensman 2023 | 2023 |  | 10.1093/ptj/pzad091 | 37669137 | Exclusion reason: Wrong intervention; |
| Mehendale 2023 | 2023 |  | 10.7759/cureus.47854 | 38021986 | Exclusion reason: Wrong intervention; |
| Koppenaal 2023 | 2023 |  | 10.2196/43034 | 37999947 | Exclusion reason: Wrong intervention; |
| Shema-Shiratzky 2022 | 2022 |  | 10.1016/j.gaitpost.2022.01.024 | 35121485 | Exclusion reason: Wrong study design; |
| Rhon 2021 | 2021 | ClinicalTrials.gov/NCT02777983 | 10.1186/s12875-021-01549-y | 34627152 | Exclusion reason: Wrong intervention; |
| Wang 2021 | 2021 |  | 10.1186/s12891-021-04409-z | 34144697 | Exclusion reason: Wrong patient population; |
| White 2021 | 2021 |  | 10.2196/22473 | 33687333 | Exclusion reason: Wrong study design; |
| Lewkowicz 2023 | 2023 |  | 10.2196/44585 | 37384379 | Exclusion reason: Wrong outcomes; |
| Cai 2017 | 2017 |  | 10.2196/mhealth.7229 | 28811270 | Exclusion reason: Wrong patient population; |
| Kaur 2023 | 2023 |  | 10.1123/jsr.2023-0005 | 37643757 | Exclusion reason: No full text; |
| PerraudinCGM 2018 | 2018 |  | 10.1159/000493277 | 32095762 | Exclusion reason: Wrong patient population; |
| Ghorbani 2020 | 2020 |  | 10.1016/j.jcm.2019.10.004 | 33192190 | Exclusion reason: Wrong comparator; |
| RodrÃ­guez-Sanz 2019 | 2019 |  | 10.3233/BMR-181260 | 30614791 | Exclusion reason: Wrong patient population; |
| Almhdawi 2020 | 2020 |  | 10.1177/0269215520937757 | 32602362 | Exclusion reason: No full text; |
| HasenÃ¶hrl 2020 | 2020 |  | 10.1007/s00508-020-01616-x | 32060724 | Exclusion reason: Wrong patient population; |
| Stenneberg 2018 | 2018 |  | 10.1016/j.msksp.2017.12.006 | 29328979 | Exclusion reason: Wrong study design; |
| YaÅŸarerÃ– 2023 | 2023 |  | 10.1080/08990220.2023.2194389 | 37026597 | Exclusion reason: No full text; |
| Priebe 2020 | 2020 |  | 10.2147/JPR.S232792 | 32547175 | Exclusion reason: Wrong study design; |
| Clement 2018 | 2018 |  | 10.2196/10422 | 29875088 | Exclusion reason: Wrong intervention; |
| Reade 2017 | 2017 |  | 10.2196/mhealth.6496 | 28341616 | Exclusion reason: Wrong patient population; |
| Sassenberg 2022 | 2022 |  | 10.2196/28913 | 35179500 | Exclusion reason: Wrong intervention; |
| Sandal 2020 | 2020 | ClinicalTrials.gov/NCT03697759 | 10.1186/s40814-020-00604-2 | 32489674 | Exclusion reason: Wrong intervention; |
| Priebe 2020 | 2020 |  | 10.2147/JPR.S260761 | 32765057 | Exclusion reason: Wrong intervention; |
| Hong 2023 | 2023 |  | 10.1016/j.ijotn.2023.101062 | 37891073 | Exclusion reason: Wrong intervention; |
| RomeroMorales 2017 | 2017 |  | 10.7717/peerj.2820 | 28070457 | Exclusion reason: Wrong patient population; |
| Nelligan 2019 | 2019 |  | 10.2196/14619 | 31628786 | Exclusion reason: Wrong study design; |
| Wijnen 2020 | 2020 | ClinicalTrials.gov/NCT03846063 | 10.2196/14139 | 32338621 | Exclusion reason: Wrong comparator; |
| Hu 2023 | 2023 |  | 10.3389/fbioe.2023.1335251 | 38264579 | Exclusion reason: Wrong intervention; |
| Beyaztas 2021 | 2021 | CN-02303800 | 10.1136/annrheumdis-2021-eular.2204 | | Exclusion reason: No full text; |
| Jain 2022 | 2022 | CN-02426827 | 10.1177/21925682221096074 | | Exclusion reason: No full text; |
| Mbada 2017 | 2017 | CN-01445998 | 10.1016/j.apmr.2017.08.143 | | Exclusion reason: No full text; |
| Pach 2020 | 2020 | CN-02230607 | 10.1177/2164956120912849 | | Exclusion reason: No full text; |
| Cana-Pino 2021 | 2021 |  | https://dx.doi.org/10.3390/ijerph18052489 | 33802528 | Exclusion reason: Wrong study design; |
| Highland 2021 | 2021 |  | https://dx.doi.org/10.1016/j.pmn.2020.10.007 | 33223470 | Exclusion reason: Wrong intervention; |
| Nishiguchi 2016 | 2016 |  | https://dx.doi.org/10.3414/ME14-01-0106 | 26391694 | Exclusion reason: Wrong study design; |
| Pichonnaz 2015 | 2015 |  | https://dx.doi.org/10.3390/s151026801 | 26506355 | Exclusion reason: Wrong patient population; |
| Rajalaxmi 2022 | 2022 | 2.02E+09 | https://dx.doi.org/10.51248/.v42i3.967 | | Exclusion reason: Wrong intervention; |
| Chauveau 2021 | 2021 | 2.02E+09 | https://dx.doi.org/10.3166/dea-2021-0156 | | Exclusion reason: No full text; |
| Jacklyn 2021 | 2021 | 6.37E+08 | https://dx.doi.org/10.4103/ija.ija_733_21 | | Exclusion reason: Wrong patient population; |
| Peleki 2016 | 2016 | 6.11E+08 | https://dx.doi.org/10.1016/j.ejvssr.2016.04.004 | | Exclusion reason: Wrong patient population; |
| Pourahmadi 2016 | 2016 | WOS:000383261200009 | 10.7717/peerj.2355 | | Exclusion reason: Wrong patient population; |
| Bedson 2019 | 2019 | WOS:000456704900001 | 10.1186/s12911-019-0741-z | | Exclusion reason: Wrong study design; |
| Khan 2023 | 2023 | WOS:000943143500003 | 10.12968/ijtr.2022.0004 | | Exclusion reason: Wrong study design; |
| Villa 2020 | 2020 | WOS:000600319400002 | 10.2196/21475 | | Exclusion reason: Wrong intervention; |
| Kawi 2022 | 2022 | WOS:000892442400005 | 10.1155/2022/8079691 | | Exclusion reason: Wrong intervention; |
| Ali 2023 | 2023 | WOS:001048870100001 | 10.1177/20552076231194544 | | Exclusion reason: Wrong study design; |
| McClincy 2021 | 2021 | WOS:000704787100022 | 10.2196/27195 | | Exclusion reason: Wrong study design; |
