# Supplementary material for: Impact of 25 Years of Mobile Health Tools for Pain Management in Patients With Chronic Musculoskeletal Pain: Systematic Review
Source: J Med Internet Res. 2024 Aug 16;26:e59358. doi: 10.2196/59358 (PMC11364951; doi:10.2196/59358)
Supplement: Multimedia Appendix 6 [file jmir_v26i1e59358_app6.docx]

**Multimedia Appendix 6. Four types of mHealth tools**

| Mobile app | This include patient portals, management systems, and other complex communication platforms that require only a commercially available smartphone. Hence, patients generally need to manually input information and proceed intervention via mobile. |
| --- | --- |
| Mobile app plus monitor | In addition to mobile, this requires additional digital activity tracker that can acting as the role of recording self-management data and usually not available on a commercial smartphone. |
| Mobile app plus wearable sensor | This usually combines mobile apps with external sensors that can record specific data in specific location (such as range of motion [ROM]) and are fully integrated into the self-management app to deliver physical therapy (PT) regimen easier. |
| Web-based mobile app plus monitor | This type includes mobile apps used by patients with monitor for participants’ goals and physical activities that can transmit records wirelessly to web-interface for viewing by both patients and health care providers. |
